# Leucine‐rich repeat kinase 2 (LRRK2) inhibitors differentially modulate glutamate release and Serine935 LRRK2 phosphorylation in striatal and cerebrocortical synaptosomes

**DOI:** 10.1002/prp2.484

**Published:** 2019-05-27

**Authors:** Daniela Mercatelli, Paolo Bolognesi, Martina Frassineti, Clarissa A. Pisanò, Francesco Longo, Derya R. Shimshek, Michele Morari

**Affiliations:** ^1^ Department of Medical Sciences Section of Pharmacology University of Ferrara Ferrara Italy; ^2^ Neuroscience Center and National Institute of Neuroscience University of Ferrara Ferrara Italy; ^3^ Department of Neuroscience Novartis Institutes for BioMedical Research Novartis Pharma AG Basel Switzerland; ^4^ Present address: Center for Neural Science New York University 4 Washington Place New York NY 10003 USA

**Keywords:** D1994S kinase‐dead, G2019S knock‐in, GSK2578215A, LRRK2, LRRK2‐IN‐1, pSer935, synaptosomes

## Abstract

Mutations in leucine‐rich repeat kinase 2 (*LRRK2*) gene have been pathogenically linked to Parkinson's disease, and pharmacological inhibition of LRRK2 is being pursued to tackle nigro‐striatal dopaminergic neurodegeneration. However, LRRK2 kinase inhibitors may have manifold actions, affecting not only pathological mechanisms in dopaminergic neurons but also physiological functions in nondopaminergic neurons. Therefore, we investigated whether LRRK2 kinase inhibitors differentially modulate dopamine and glutamate release from the mouse striatum and cerebral cortex. Spontaneous and KCl‐evoked [^3^H]‐dopamine and glutamate release from superfused synaptosomes obtained from wild‐type and LRRK2 knock‐out, kinase‐dead or G2019S knock‐in mice was measured. Two structurally unrelated inhibitors, LRRK2‐IN‐1 and GSK2578215A, were tested. LRRK2, phosphoSerine1292 and phosphoSerine935 LRRK2 levels were measured in all genotypes, and target engagement was evaluated by monitoring phosphoSerine935 LRRK2. LRRK2‐IN‐1 inhibited striatal glutamate but not dopamine release; GSK2578215A inhibited striatal dopamine and cortical glutamate but enhanced striatal glutamate release. LRRK2‐IN‐1 reduced striatal and cortical phosphoSerine935 levels whereas GSK2578215A inhibited only the former. Neither LRRK2 inhibitor affected neurotransmitter release in LRRK2 knock‐out and kinase‐dead mice; however, they facilitated dopamine without affecting striatal glutamate in G2019S knock‐in mice. GSK2578215A inhibited cortical glutamate release in G2019S knock‐in mice. We conclude that LRRK2‐IN‐1 and GSK2578215A modulate exocytosis by blocking LRRK2 kinase activity, although their effects vary depending on the nerve terminal examined. The G2019S mutation unravels a dopamine‐promoting action of LRRK2 inhibitors while blunting their effects on glutamate release, which highlights their positive potential for the treatment of PD, especially of LRRK2 mutation carriers.

**Abbreviations**
DA: Dopamine
ECL: Enhanced chemiluminescence luciferase
GLU: Glutamate
IN‐1: LRRK2‐IN‐1
KD: Kinase dead
KI: Knock‐in
KO: Knock‐out
LRRK2: Leucine‐rich repeat kinase 2
PD: Parkinson's disease
pSer1292: Phosphoserine 1292
pSer935: Phosphoserine 935
ROC: Ras of complex
WT: Wild‐type

## 1 INTRODUCTION

Leucine‐rich repeat kinase 2 (LRRK2) is a 2527 amino acid, multifunctional protein endowed with a kinase domain and a Ras of complex (ROC) domain with GTPase activity, surrounded by protein‐protein interaction domains.^1^, ^2^ Mutations in the *LRRK2* gene are associated with familial late‐onset 3, 4 and sporadic 5 Parkinson's disease (PD). To further emphasize the role of LRRK2 in idiopathic PD, genome‐wide studies have revealed LRRK2 to be associated with an increased risk of PD.^6^ At present, at least six mutations, that is, the G2019S and I2020T in the kinase domain, the R1441C/G/H and in the ROC domain, and the Y1669C in the CoR domain, have been consistently shown to be pathogenic.^7^ Limited evidence for the p.N1437H mutation in the ROC domain has also been presented.^8^ In particular, expression of the G2019S mutation is associated with enhanced kinase activity and neurodegeneration in vitro.^9^, ^10^ This has boosted the development of LRRK2 kinase inhibitors as novel disease modifying agents, able to attenuate nigro‐striatal dopamine (DA) neuron loss in PD.^11^, ^12^, ^13^ Nonetheless, the possibility that LRRK2 inhibitors interfere with cell homeostatic functions, in the same or different neuronal populations or tissues, exists,^14^ which raises safety issues about this class of compounds. Thus, comparing the effects of LRRK2 inhibitors on different neuronal populations, in both wild‐type (WT) and LRRK2 mutant expressing animals, is mandatory.

Among the various cellular functions modulated by LRRK2, exocytosis appears attractive because LRRK2 can regulate neurotransmitter release via multiple routes,^15^ for example, by modulating vesicle mobility and trafficking,^16^, ^17^, ^18^ SNARE protein assembly,^18^, ^19^ and presynaptic Ca^++^ entry.^20^ Given the pathogenic role of LRRK2 in PD, a wealth of studies focused on in vivo and in vitro DA release using LRRK2 knock‐out (KO) mice,^21^, ^22^, ^23^ G2019S 24, 25 or R1441C 26 knock‐in (KI) mice, hG2019S or hR1441G overexpressing mice 27, 28, 29, 30, 31 or rats.^32^, ^33^ Fewer studies attempted to address the role of LRRK2 in the release of other neurotransmitters, focusing specifically on in vitro glutamate (GLU) release in the cortex 16, 27, 34 and hippocampus.^35^ None of these studies, however, performed a simultaneous analysis of DA and GLU release within a specific or different brain areas, to investigate whether LRRK2 control of neurotransmitter release is similar across different subpopulations of nerve terminals. Moreover, only a few studies employed more than one LRRK2 kinase inhibitor, leaving to speculation whether these molecules, in addition to sharing class‐specific properties have peculiar effects. In fact, it has been previously shown that pharmacological blockade of kinase activity results in rapid dephosphorylation of LRRK2 at Ser935, an index of kinase activity inhibition and disturbance of LRRK2 binding to 14‐3‐3,^36^ followed by delayed LRRK2 degradation through the ubiquitin‐proteasome pathway.^37^ LRRK2 inhibitors might have a different ability to influence such mechanisms, as shown in primary astrocytes where only GSK2578215A 38 among a panel of 6 different LRRK2 inhibitors, was able to induce protein destabilization.^37^ This would suggest that LRRK2 inhibitors might have not only a different potency but also a different mode of interaction with LRRK2 kinase pocket. In fact, while the ability of LRRK2‐IN‐1 (IN‐1) to inhibit LRRK2 was dramatically reduced (by 190‐folds) in A2016T mutants,^39^ that of GSK2578215A was minimally affected (7‐folds).^38^

For these reasons, in this study we investigated whether two structurally unrelated LRRK2 kinase inhibitors, such as IN‐1 and GSK2578215A, differentially affect the spontaneous and KCl‐evoked [^3^H]‐DA and GLU release in superfused synaptosomes from the mouse striatum and cerebral cortex. Synaptosomes represent a basic preparation of nerve endings, suitable for studying exocytosis since they preserve the release machinery (ATP‐ and Ca^++^‐dependent release), express membrane and vesicular transporters, and expose autoreceptors. In this preparation, the KCl‐evoked neurotransmitter efflux relies on exocytotic Ca^++^‐ dependent and, partly, Na^+^‐dependent mechanisms, whether spontaneous efflux is essentially non exocytotic.^40^ Moreover, the superfusion conditions adopted in this study ensure a rapid removal of the neurotransmitter from the medium, thus minimizing neurotransmitter uptake and autoreceptor activation,^41^, ^42^ which might confound the effect of the depolarizing stimulus and LRRK2 inhibitors on exocytosis. The effects of IN‐1 and GSK2578215A were first investigated in synaptosomes from WT mice, then in synaptosomes from mice with constitutive deletion of LRRK2 (KO mice) or knock‐in for the LRRK2 D1994S kinase‐dead mutation (KD mice) to confirm their pharmacological specificity. Since LRRK2 inhibitors are expected to be used in G2019S carriers first, their effects were also investigated in synaptosomes from mice expressing the LRRK2 kinase‐enhancing G2019S mutation (G2019S KI mice).^21^, ^24^, ^43^ Finally, LRRK2 protein levels and kinase activity (pSer1292 and pSer935 levels) were measured in striatal and cortical tissue lysates and synaptosomes, and target engagement of LRRK2 inhibitors assessed.

## 2 MATERIALS AND METHODS

### 2.1 Animals

Experiments were performed in accordance with the ARRIVE guidelines. Experimenters were blinded to treatments. Three‐month‐old male mice (25‐30 g), backcrossed on a C57BL/6J background, were used in the study. Homozygous LRRK2 KO mice (founders obtained from Mayo Clinic, Jacksonville, FL, USA),^22^ KD and G2019S KI mice (founders obtained from Novartis Institutes for BioMedical Reserch, Novaris Pharma AG, Basel, Switzerland) 21 were used. A colony of nontransgenic wild‐type (WT) mice was initially set from heterozygous breeding of G2019S KI LRRK2 mice, then control WT male mice used in all experiments were obtained from homozygous breeding. Colonies were grown in the *vivarium* of the Department of Medical Sciences, at the University of Ferrara, with free access to food (4RF21 standard diet; Mucedola, Settimo Milanese, Milan, Italy) and water, and kept under regular lighting conditions (12 h dark/light cycle). Animals were housed in groups of 5 for a 55 × 33x20 cm polycarbonate cage (Tecniplast, Buguggiate, Varese, Italy) with a Scobis Uno bedding (Mucedola, Settimo Milanese, Milan, Italy) and environmental enrichments. Experimental protocols were approved by the Italian Ministry of Health (license 134/2017‐PR).

### 2.2 Synaptosome preparation

Synaptosomes were prepared as previously described.^42^, ^44^ Mice were anesthetized and sacrificed via cervical dislocation. Striatum or cerebral cortex (fronto‐temporal areas) from each mouse were homogenized in ice‐cold 0.32 mol L^−1^ sucrose, HEPES 5 mmol L^‐1^, EDTA 30 µmol L^‐1^ and MgSO_4_ 0.52 mmol L^‐1^ (pH 7.4) with a Teflon‐glass homogenizer and centrifuged at 800*g* for 10 minutes at 4°C. The supernatant was then centrifuged at 11 000*g* for 20 minutes at 4°C, the pellet resuspended in 1.5 or 0.6 mL oxygenated (95% O_2_, 5% CO_2_) Krebs solution (mM: NaCl 118.5, KCl 4.7, CaCl_2_ 1.2, MgSO_4_ 1.2, KH_2_PO_4_ 1.2, NaHCO_3_ 25, glucose 10), and processed for (i) release experiments or (ii) Western blot analysis, respectively.

### 2.3 Release studies

Synaptosomes were incubated at 36.5°C with 50 nmol L^−1^ [^3^H]‐DA (specific activity 40 Ci mmol^−1^; Perkin‐Elmer, Boston, MA) for 25 minutes, after which 12 mL of preoxygenated Krebs were added.^24^, ^40^ One milliliter aliquots of the suspension (~0.35 mg protein) were slowly injected into nylon syringe filters (outer diameter 13 mm, 0.45 μmol L^−1^ pore size, internal volume ~100 μL; Teknokroma, Barcelona, Spain), maintained at 36.5°C in a thermostatic bath and superfused (0.4 mL min^−1^) with a precarbogenated Krebs solution. Filters were washed for 20 minutes, after which sample collection was started (every 3 minutes). After three basal samples, a 90 seconds pulse of 15 mmol L^−1^ KCl was delivered (KCl 10 mmol L^−1^ was also applied when head‐to‐head comparison experiments among genotypes were performed) and sample collection continued for further 16.5 minutes. The effect of kinase inhibitors was evaluated both on spontaneous efflux and KCl‐stimulated neurotransmitter outflow. Inhibitors were added to the perfusion medium 9 minutes before the pulse of KCl, and maintained until the end of the experiment. Radioactivity in the samples and in the filters was measured using a Perkin Elmer Tri Carb 2810 TR scintillation counter.

Endogenous GLU levels in the samples were measured by HPLC coupled with fluorometric detection.^40^ Thirty microliters of o‐phthaldialdehyde/mercaptoethanol reagent were added to 30 μL aliquots of sample, and 50 μL of the mixture was automatically injected (Triathlon autosampler; Spark Holland, Emmen, Netherlands) onto a 5‐C18 Hypersil ODS column (3 × 100 mm; Thermo‐Fisher, USA) perfused at a flow rate of 0.48 mL min^−1^ (Jasco PU‐2089 PLUS; Jasco, Tokyo, Japan) with a mobile phase containing 0.1 M sodium acetate, 10% methanol and 2.2% tetrahydrofuran (pH 6.5). GLU was detected by means of a fluorescence spectrophotometer FP‐2020 Plus (Jasco, Tokyo, Japan) with the excitation and the emission wavelengths set at 370 and 450 nm, respectively. The limit of detection for GLU was ~1 nmol L^−1^.

### 2.4 Western blot analysis

Synaptosomes were incubated with vehicle or increasing concentrations of LRRK2 inhibitors (0.001‐3 μmol L^−1^ IN‐1, 0.1‐10 μmol L^−1^ GSK‐2578215A) for 3, 12 and/or 30 minutes. At the end of incubation, the suspension was centrifuged at 11 000 *g* for 5 minutes at 4°C. The pellet was then solubilized and homogenized in lysis buffer (RIPA buffer, protease and phosphatase inhibitor cocktail) and centrifuged at 18 000 *g* for 15 minutes at 4°C. Supernatants were collected and total protein levels were quantified using the bicinchoninic acid protein assay kit (Thermo Scientific). Thirty micrograms of protein per sample were separated by SDS‐PAGE, transferred onto polyvinyldifluoride membrane and tested for the following primary antibodies: anti‐pSer935 LRRK2 (Abcam, ab133450, 1:300), anti‐pSer1292 (Abcam, ab203181, 1:300) and anti‐α‐tubulin (Merck Millipore 04‐1117, 1:50,000). Horseradish peroxidase‐linked secondary antibody (Merck Millipore, goat anti‐rabbit IgG HRP‐conjugate 12‐348, 1:4000) was then used and immunoreactive proteins were visualized by enhanced chemiluminescence luciferase (ECL) detection kit (Pierce^™^ BCA Protein Assay Kit, Thermo Scientific or ECL+, GE Healthcare). Images were acquired and quantified using the ChemiDoc MP System and the ImageLab Software (Bio‐Rad). Membranes were then stripped and re‐probed with rabbit anti‐LRRK2 (Abcam, ab133474, 1:1000). Data were analyzed by densitometry; the optical density of pSer935 LRRK2 and pSer1292 LRRK2 was normalized to total LRRK2 whereas total LRRK2 was normalized to α‐tubulin levels. To minimize experimental variability, each blot was replicated twice, and data averaged.

### 2.5 Data presentation and statistical analysis

**Figure 1:**
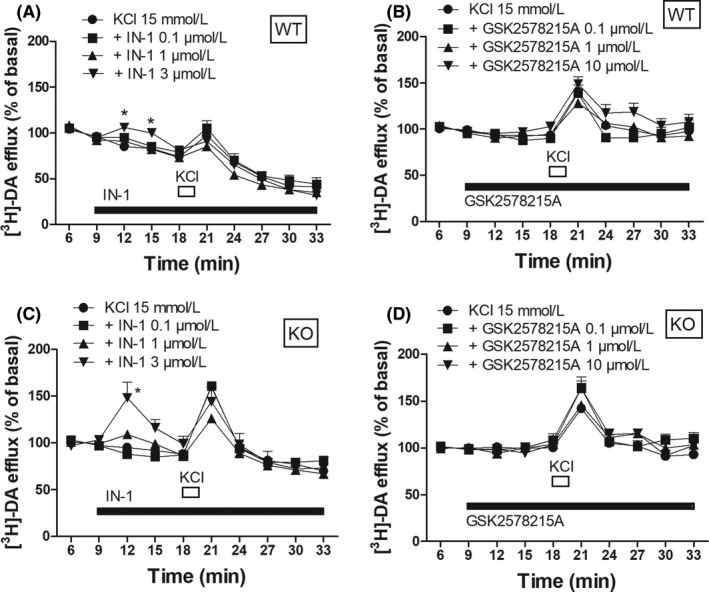
[^3^H]‐DA efflux in striatal synaptosomes from WT (A‐B) and KO (C‐D) mice superfused with IN‐1 (A,C) or GSK2578215A (B,D), and stimulated with 15 mmol L^−1^ KCl (90 seconds). Data are means ± SEM of 14 (panels A‐B), 15 (panel C) and 12 (panel D) determinations per group, and are expressed as fractional release (FR). *P < 0.05, different from KCl 15 mmol L^−1^ (two‐way ANOVA followed by the Bonferroni test)

**Figure 2:**
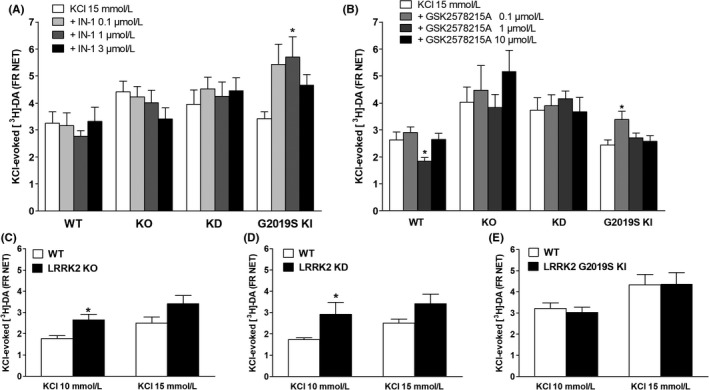
[^3^H]‐DA release in striatal synaptosomes from WT, KO, KD and G2019S KI mice superfused with IN‐1 (A) or GSK2578215A (B), and stimulated with 15 mmol L^−1^ KCl (90 seconds). Head‐to‐head comparisons of 15 mmol L^−1^ or 10 mmol L^−1^ KCl‐evoked [^3^H]‐DA release in KO, KD and G2019S KI mice and respective wild‐type controls (C‐E). Data are means ± SEM of n determinations per group (see details below) and are expressed as net fractional release (FR NET). Determinations per group: panel 2A, n = 15 (WT and KO), n = 12 (KD), n = 18 (KI; n = 9 for the 3 μmol L^−1^ group); panel 2B, n = 15 (WT), n = 12 (KO), n = 9 (KD) n = 18 (KI); panel 2C, n = 17; panels 2D and 2E, n = 12. *P* < 0.05, different from KCl 15 mmol L^−1^ (A‐B) or KCl in WT mice (C‐E) (Student's *t* test, 2‐tailed for unpaired data, or one‐way ANOVA followed by the Bonferroni test)

**Figure 3:**
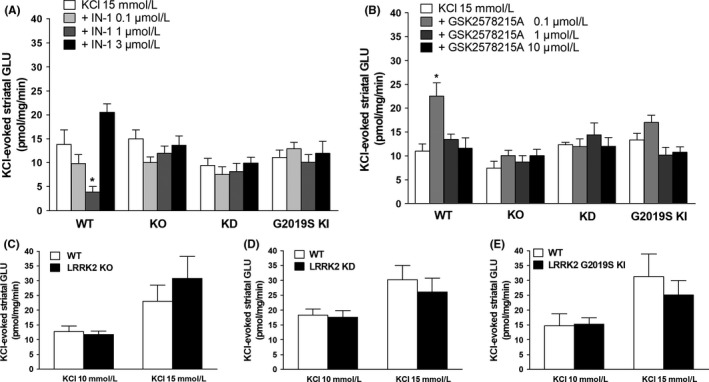
GLU release in striatal synaptosomes from WT, KO, KD and G2019S KI mice superfused with IN‐1 (A) or GSK2578215A (B), and stimulated with 15 mmol L^−1^ KCl (90 seconds). Head‐to‐head comparisons of 15 mmol L^−1^ or 10 mmol L^−1^ KCl‐evoked GLU release in KO, KD and G2019S KI mice and respective wild‐type controls (C‐E). Data are means ± SEM of n determinations per group (see details below) and are expressed as pmol mg min^−1^. Determinations per group: panel A, WT (n = 8), KO n = 15, KD (n = 9; one outlier in the 0.1 μmol L^−1^ group), KI (n = 8); panel B, WT (n = 12; one outlier in the 0.1 μmol L^−1^ group), KO n = 12, KD n = 9, KI n = 15 (control) n = 18 (0.1 μmol L^−1^), n = 17 (1 and 10 μmol L^−1^; one outlier in each group), panel C (n = 8 KCl 10 mmol L^−1^, and n = 9 KCl 15 mmol L^−1^), panel D (n = 12), panel E (n = 8). *P < 0.05, different from KCl 15 mmol L^‐1^ (Student's *t* test, two‐tailed for unpaired data, or one‐way ANOVA followed by the Bonferroni test)

**Figure 4:**
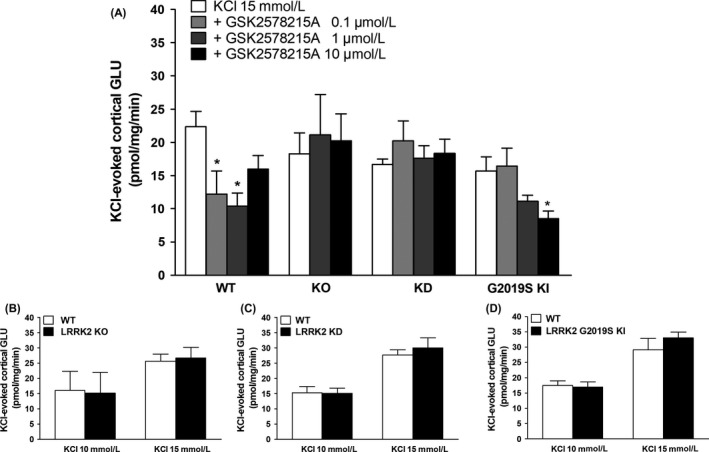
GLU release in cortical synaptosomes from WT, KO, KD and G2019S KI mice superfused with GSK2578215A (A) and stimulated with 15 mmol L^−1^ KCl (90 seconds). Head‐to‐head comparisons of 15 mmol L^−1^ or 10 mmol L^−1^ KCl‐evoked GLU release in KO, KD and G2019S KI mice and respective wild‐type controls (B‐D). Data are means ± SEM of n determinations per group (see details below) and are expressed as pmol mg min^−1^. Determinations per group: panel A, WT (n = 8, n = 6 in the 10 μmol L^−1^ group), KO n = 12, KD n = 9, KI n = 11 (n = 9 in the 10 μmol L^−1^ group); panel B, n = 5 (KCl 10 mmol L^−1^), n = 11 (KCl 15 mmol L^−1^); panel C n = 9; panel D n = 9. *P < 0.05, different from KCl 15 mmol L^−1^ (one‐way ANOVA followed by the Bonferroni test)

The data and statistical analysis comply with the recommendations on experimental design and analysis in pharmacology.^45^ Neurochemical data are means ± SEM (standard error of the mean) of n determinations per group. Tritium efflux (Figure 1) was calculated as fractional release (FR, ie tritium efflux expressed as percentage of the tritium content in the filter at the onset of the corresponding collection period) whereas KCl‐evoked tritium overflow (Figure 2) was calculated as FR NET, that is, tritium overflow as percent of the tritium content in the filter at the onset of the corresponding collection period. GLU release (Figures 3 and 4) was expressed as pmol mg protein^−1^. Western blot data have been presented as ratio between pSer935 or pSer1292 and total LRRK2, or between total LRRK2 and α‐tubulin (housekeeper). Statistical analysis on neurochemical and biochemical data was performed (Prism software; San Diego, CA) by two‐way or one‐way ANOVA followed the Bonferroni test for multiple comparisons. When only two groups were compared, the Student's *t* test, 2‐tailed for unpaired data, was used. Outliers were identified using the Outlier calculator freely available in Graphpad Prism software. *P*‐values < 0.05 were considered to be statistically significant.

### 2.6 Materials

IN‐1 and GSK2578215A were purchased from Tocris Bioscience (Bristol, UK). Both compounds were dissolved in DMSO to 10 mmol L^−1^, then diluted with Krebs at the tested concentrations.

### 2.7 Nomenclature of Targets and Ligands

Key protein targets and ligands in this article are hyperlinked to corresponding entries in http://www.guidetopharmacology.org, the common portal for data from the IUPHAR/BPS Guide to PHARMACOLOGY,^46^ and are permanently archived in the Concise Guide to PHARMACOLOGY 2015/16.^47^

## 3 RESULTS

### 3.1 Striatal DA release

To investigate whether LRRK2 regulates DA release, we first monitored whether LRRK2 inhibitors modulate the spontaneous and KCl‐evoked [^3^H]‐DA efflux from striatal WT synaptosomes. The time‐course of [^3^H]‐DA response to a 90 seconds pulse of 15 mmol L^−1^ KCl is shown in Figure 1. The KCl pulse evoked a rapid and transient elevation of [^3^H]‐DA efflux, that returned to baseline after stimulus withdrawal (Figure 1A; treatment *F*_3,9_ = 6.437 *P* = 0.0003, time *F*_9,390_ = 93.56 *P* < 0.0001, time X treatment interaction *F*_27,390_ = 1.72, *P* = 0.014). IN‐1 superfused 9 minutes before the KCl pulse, caused a transient elevation of spontaneous [^3^H]‐DA efflux at 3 μmol L^−1^ (Figure 1A) but failed to alter the KCl‐evoked striatal [^3^H]‐DA release (Figure 2A). Different from IN‐1, GSK2578215A was ineffective on spontaneous [^3^H]‐DA efflux (Figure 1B) but inhibited the KCl‐evoked [^3^H]‐DA release (Figure 2B; treatment *F*_3,56_ = 3.247 *P* = 0.0061). The inhibition was mild (30%) and only observed at 1 μmol L^−1^.

The specificity of the IN‐1 and GSK2578215A effects was verified in LRRK2 KO mice. As a preliminary approach, a comparison of [^3^H]‐DA release in WT and KO mice was made. No difference in spontaneous tritium efflux between genotypes was observed (Table 1), and 15 mmol L^−1^ KCl tended to be more effective in KO than WT mice (Figure 2C). We therefore used a milder stimulation and found that 10 mmol L^−1^ KCl was significantly more effective (+46%) in KO than WT mice (Figure 2C, *t* = 3.066 *df* = 32). IN‐1 caused an increase in [^3^H]‐DA efflux at 3 μmol L^−1^ also in KO mice (Figure 1C; treatment *F*_3,9_ = 0.46 *P* = 0.69; time *F*_9,504_ = 43.39 *P* < 0.0001, time X treatment interaction *F*_27,504_ = 2.801 *P* < 0.0001), being ineffective on stimulus‐evoked DA release (Figure 2A). Conversely, GSK2578215A was ineffective in KO mice (Figure 1D and Figure 2B). To confirm the involvement of LRRK2 activity in the effects of IN‐1 and GSK2578215A, we tested both compounds in KD mice. Again, [^3^H]‐DA release in KD and WT mice was first compared. No difference in spontaneous efflux between genotypes was observed (Table 1). As observed in KO mice, 10 mmol L^−1^ KCl induced a larger tritium outflow in KD mice (Figure 2D, *t* = 2.130 *df* = 22). Likewise, both IN‐1 and GSK2578215A were ineffective in KD mice (Figure 2A–B).

**Table 1:** Spontaneous neurotransmitter release in mouse striatal and cortical synaptosomes. Data were obtained under head‐to‐head comparisons between LRRK2 KO, KD, and G2019S KI mice with respective wild‐type controls

Genotype	[^3^H]‐DA striatum (FR)		GLU striatum (nmol L^−1^)		GLU cortex (nmol L^−1^)	
		n		n		n
LRRK2 KO	8.82 ± 0.024	28	17.31 ± 1.26	18	18.87 ± 1.45	12
WT	8.84 ± 0.23	28	17.99 ± 1.16	18	19.56 ± 1.39	12
KD	7.38 ± 0.32%	24	19.51 ± 1.63	24	23.01 ± 1.65	18
WT	6.68 ± 0.22%	24	20.48 ± 0.97	24	22.35 ± 1.61	18
G2019S KI	5.31 ± 0.19%	24	19.48 ± 2.10	18	17.54 ± 1.42	18
WT	5.86 ± 0.26%	24	18.68 ± 1.15	18	17.29 ± 1.45	18

Data are expressed as fractional release (FR, [^3^H]‐DA) or absolute concentrations (nmol L^‐1^, glutamate, GLU), and are mean ± SEM of n determinations per group, as detailed in the table

Finally, we evaluated the impact of LRRK2 kinase inhibition on [^3^H]‐DA release in G2019S KI mice. No difference in spontaneous (Table 1) or stimulus‐evoked (Figure 2E) [^3^H]‐DA release was detected between G2019S KI and WT mice. IN‐1 elevated the KCl‐evoked striatal [^3^H]‐DA release at 0.1 μmol L^−1^ (~60%) but not higher concentrations (Figure 2A; treatment *F*_3,59_ = 2.915 *P* = 0.0416). Such profile was replicated by GSK2578215A, which facilitated (~40%) striatal [^3^H]‐DA release at 0.1 μmol L^−1^ but not higher concentrations (Figure 2B; treatment *F*_3,68_ = 3.457 *P* = 0.0211).

### 3.2 Striatal GLU release

LRRK2 kinase inhibitors induced dramatic effects on striatal GLU release in WT synaptosomes. IN‐1 was ineffective on spontaneous GLU efflux (not shown), but markedly reduced (~75%) the KCl‐evoked striatal GLU release from WT synaptosomes at 1 μmol L^−1^ (Figure 3A; treatment *F*_3,28_ = 11.35 *P* < 0.0001), the effect being reversed at higher concentrations. GSK2578215A was also ineffective on spontaneous GLU efflux (not shown) but, contrary to IN‐1, elevated the KCl‐evoked striatal GLU release in WT synaptosomes (Figure 3B; treatment *F*_3,43_ = 5.812 *P* = 0.0020). A robust potentiation (~100%) was observed at 0.1 μmol L^−1^ but not higher concentrations. We also separately tested GSK2578215A 0.01 μmol L^−1^ and found it was unable to modify the K^+^‐evoked GLU release (KCl/GSK2578215A 12.05 ± 2.4 mg prot min^−1^ vs KCl alone 9.07 ± 1.39 mg prot min^−1^, n = 6 each). The specificity of these effects was verified in KO and KD synaptosomes. Spontaneous (Table 1) or KCl‐evoked striatal GLU release (Figure 3C‐D) was similar in WT, KO and KD synaptosomes. Both IN‐1 and GSK2578215A were ineffective in KO and KD synaptosomes (Figure 3A‐B). Finally, IN‐1 and GSK2578215A were tested in G2019S KI synaptosomes (Figure 3A‐B). Spontaneous (Table 1) or stimulus‐evoked (Figure 3E) striatal GLU release was similar in WT and G2019S KI synaptosomes. IN‐1 and GSK2578215A were ineffective in G2019S KI synaptosomes (Figure 3A‐B).

### 3.3 Cortical GLU release

We previously reported that IN‐1 inhibited cortical GLU release in a LRRK2‐specific way.^34^ Similar to IN‐1, GSK2578215A markedly inhibited the KCl‐evoked cortical GLU release in WT synaptosomes (treatment *F*_3,26_ = 4.391 *P* = 0.0120), being effective at 0.1 μmol L^−1^ and 1 μmol L^−1^ (−45% and −54%, respectively; Figure 4A). To prove the LRRK2‐specificity, KO and KD synaptosomes were used. No differences between genotypes were observed in spontaneous (Table 1) and stimulus‐evoked (Figure 4B‐C) cortical GLU release. GSK2578215A was ineffective in LRRK2 KO and KD synaptosomes (Figure 4A). Finally, the impact of GSK2578215A in LRRK2 G2019S KI synaptosomes was investigated. Spontaneous (Table 1) and stimulus‐evoked (Figure 4D) cortical GLU release were similar between G2019S KI and WT synaptosomes. GSK2578215A inhibited the stimulus‐evoked cortical GLU release in G2019S KI synaptosomes (Figure 4A; *F*_3,38_ = 3.733 *P* = 0.0191), although less potently than in WT synaptosomes, being effective only at 10 μmol L^−1^.

### 3.4 Ser1292 LRRK2 and Ser935 LRRK2 phosphorylation

**Figure 5:**
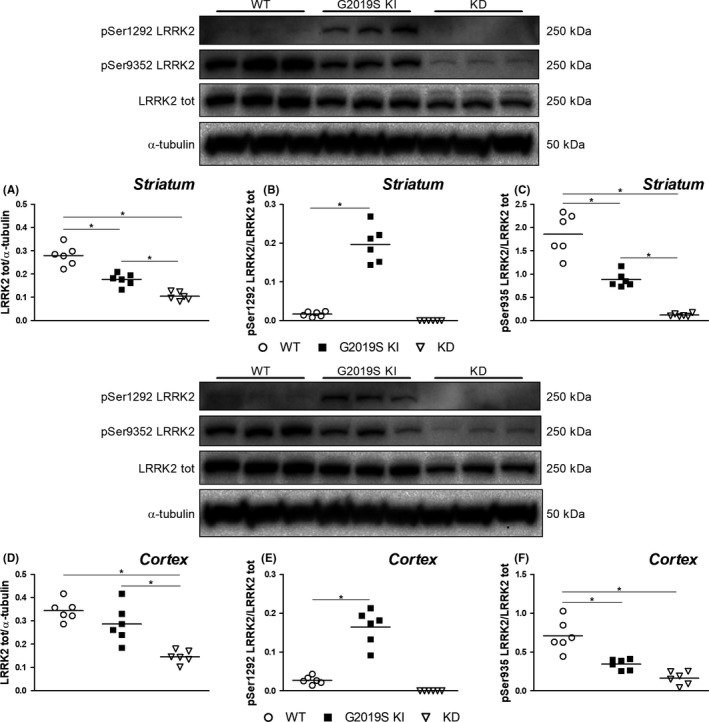
Representative immunoblots and total LRRK2 levels (A,D), pSer1292 (B,E), and pSer935 (C,F) levels in striatal and cortical synaptosomes from WT, KD and G2019S KI mice. Data are means of 6 determinations per genotype. Each determination represents the mean of 2 technical replicates. *P < 0.05 (Student's *t* test or one‐way ANOVA followed by the Bonferroni test)

In the attempt to demonstrate the engagement of synaptosomal LRRK2 by kinase inhibitors, preliminary analysis of endogenous LRRK2 levels and kinase activity was performed in striatum and cerebral cortex. Kinase activity was evaluated by quantifying pSer1292 and pSer935 LRRK2 levels, first in tissue lysates, to allow a comparison with published studies, then in synaptosomes to specifically investigate presynaptic LRRK2. In striatal lysates, LRRK2 levels were found to be lower in G2019S KI (−30%) and KD (−68%) mice compared to WT mice (*F*_2,15_ = 31.50, *P* < 0.0001), and undetectable in KO mice (Supplementary Figure 1A). pSer1292 levels were 18‐fold elevated compared to WT mice (*t* = 11.69, *df* = 10) but undetectable in both KD and KO mice (Figure S1B). Conversely, pSer935 levels were reduced by 60% in G2019S KI mice and by 89% in KD mice (*F*_2,15_ = 141.9 *P* < 0.0001; Figure S1C). The overall pattern in cerebral cortex lysates showed notable differences compared to striatum. In fact, cortical LRRK2 levels were unchanged in G2019S KI mice, and 50% lower in KD mice than WT mice (*F*_2,15_ = 16.69, *P* = 0.0002; Figure S1D), pSer1292 levels were 6‐fold elevated in G2019S KI mice compared to WT mice (*t* = 5.59, *df* = 10, *P* = 0.002; Figure S1E), whereas pSer935 levels were reduced by 36% in G2019S KI mice and by 60% in KD mice (*F*_2,15_ = 26.23, *P* < 0.0001; Figure S1F). LRRK2 profile in synaptosomes was similar to that found in tissue lysates. Total synaptosomal LRRK2 levels were reduced in the striatum of G2019S KI mice (−36%) and KD mice (−63%) compared to WT mice (*F*_2,15_ = 47.55, *P* < 0.0001; Figure 5A). pSer1292 levels were markedly increased in striatal synaptosomes of G2019S KI mice (12‐fold; *t* = 9.31 *df* = 10) but undetectable in synaptosomes from KD mice (Figure 5B) whereas pSer935 levels were reduced by 53% in G2019S KI mice and almost suppressed (−95%) in KD mice (*F*_2,15_ = 62.08 *P* < 0.0001; Figure 5C). Total LRRK2 levels were unchanged in cerebrocortical synaptosomes from G2019S KI mice whereas a 57% reduction was observed in cerebrocortical synaptosomes from KD mice (*F*_2,15_ = 20.46, *P* < 0.0001; Figure 5D). pSer1292 levels were 6‐fold elevated in cerebrocortical synaptosomes from G2019S KI mice (*t* = 7.36, *df* = 10) but undetectable in cerebrocortical synaptosomes from KD mice (Figure 5E) whereas pSer935 levels were reduced by 50% in cerebrocortical synaptosomes from G2019S KI mice and by 78% in KD mice (*F*_2,15_ = 26.33 *P* < 0.0001; Figure 5F).

**Figure 6:**
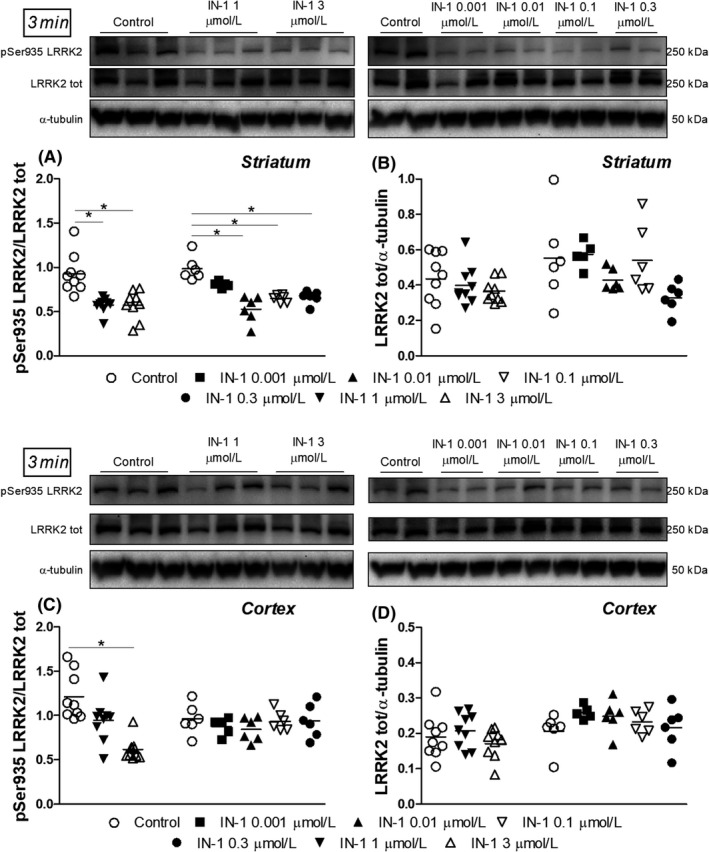
Representative immunoblots, pSer935 (A,C) and total (B,D) LRRK2 levels in striatal and cortical synaptosomes from WT mice superfused with IN‐1 (0.001‐3 μmol L^−1^) for 3 minutes. Data are means of 9 determinations (experiment with IN‐1 1‐3 μmol L^−1^) or 6 determinations (experiment with IN‐1 0.001‐3 μmol L^−1^) per group. Each determination represents the mean of 2 technical replicates. *P < 0.05 (one‐way ANOVA followed by the Bonferroni test)

**Figure 7:**
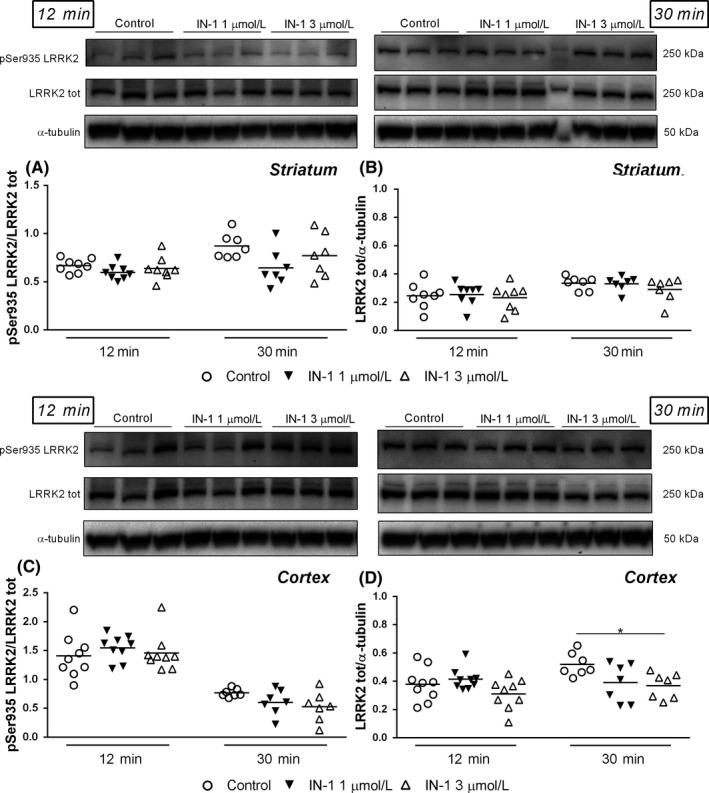
Representative immunoblots, pSer935 (A,C) and total (B,D) LRRK2 levels in striatal and cortical synaptosomes from WT mice superfused with IN‐1 (1 and 3 μmol L^−1^) for 12 or 30 min. Data are means of 9 determinations (12 minutes) or 7 determinations (30 minutes) per group. Each determination represents the mean of 2 technical replicates. *P < 0.05 (one‐way ANOVA followed by the Bonferroni test)

**Figure 8:**
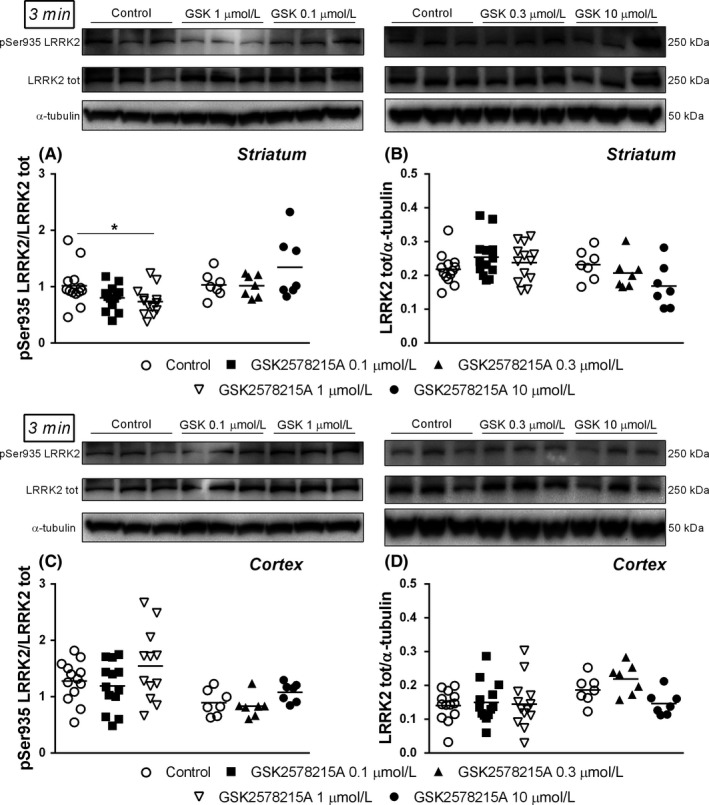
Representative immunoblots, pSer935 (A,C) and total (B,D) LRRK2 levels in striatal and cortical synaptosomes from WT mice superfused with GSK2578215A (experiment with 0.1‐10 μmol L^−1^) for 3 minutes. Data are means of 13 determinations (experiment with GSK2578215A 0.1 and 1 μmol L^−1^) or 7 determinations (experiment with GSK2578215A 0.3 and 10 μmol L^−1^) per group. Each determination represents the mean of 2 technical replicates. *P < 0.05 (one‐way ANOVA followed by the Bonferroni test)

Given the very low levels of pSer1292 in cerebrocortical synaptosomes from WT mice, we therefore decided to evaluate LRRK2 engagement by measuring pSer935 levels in striatal and cortical synaptosomes acutely treated with IN‐1 and GSK2578215A. A 3 minutes application with high concentrations of IN‐1 (1 and 3 μmol L^−1^) was first evaluated (Figure 6). Both concentrations reduced pSer935 levels in striatal synaptosomes by ~40% (*F*_2,24_ = 13.62, *P* < 0.0001; Figure 6A). To draw a complete concentration‐response curve, lower concentrations (0.001‐0.3 μmol L^−1^) were then tested. IN‐1 was effective also within this concentration range (*F*_4,24_ = 18.11, *P* < 0.0001), causing a nonsignificant 18% inhibition at 0.001 μmol L^−1^ and a 35%‐45% inhibition at 0.01‐0.3 μmol L^−1^ (Figure 6A). IN‐1 reduced pSer935 levels also in cerebrocortical synaptosomes (*F*_2,24_ = 16.13, *P* < 0.0001) but only at 3 μmol L^−1^ (50%) being the reduction observed at 1 μmol L^−1^ (22%) not significant (Figure 6C). Different from striatum, IN‐1 was ineffective at lower concentrations in cerebral cortex (*F*_4,24_ = 0.65, *P* = 0.62; Figure 6C). IN‐1 did not reduce total LRRK2 protein levels in striatal and cerebrocortical synaptosomes at 3 minutes (Figure 6B,D). The effect of IN‐1 1 μmol L^−1^ and 3 μmol L^−1^ was evaluated after longer application times. IN‐1 failed to affect pSer935 levels when applied for 12 minutes or 30 minutes (Figure 7A). Moreover, IN‐1 did not affect total LRRK2 levels after 12 minutes application (Figure 7B,D) whereas a significant 30% inhibition was observed after 30 minutes application of 3 μmol L^−1^ IN‐1 in cerebrocortical synaptosomes (Figure 7D; *F*_2,18_ = 4.13 *P* = 0.0324). GSK2578215A (0.1‐10 μmol L^−1^) only partially replicated the IN‐1 profile. Indeed, after 3 minutes application it reduced by 28% pSer935 levels in striatum at 1 μmol L^−1^ (*F*_2,36_ = 3.519 *P* = 0.0402; Figure 8A) but was ineffective on pSer935 levels in cortex at any concentrations (Figure 8C). Moreover, no effect of GSK2578215A on LRRK2 levels in striatal or cortical synaptosomes was observed (Figure 8B,D). Longer application times of GSK2578215A were evaluated (Figure S2), and similar to IN‐1, GSK2578215A failed to alter striatal (Figure S2A) and cortical (Figure S2C) pSer935 levels after 12 minutes or 30 minutes. Different from IN‐1, GSK2578215A did not alter LRRK2 protein levels at any concentrations (Figure S2B,D).

## 4 DISCUSSION

A synaptosomal preparation was used to investigate the role of LRRK2 in neurotransmitter release ([^3^H]‐DA and GLU) in different brain areas (striatum and cerebral cortex), using two different LRRK2 inhibitors (IN‐1 and GSK2578215A) and different mouse genotypes, that is, WT, KO, KD, and G2019S KI mice (Table 2). LRRK2 inhibitors did not change (IN‐1) or slightly inhibited (GSK2578215A) the stimulus‐evoked DA release in WT synaptosomes suggesting that under normal conditions LRRK2 does not strongly control exocytotic DA release. This contrasts with the potentiation of stimulus‐evoked tritium release consistently found in striatal KO and KD synaptosomes, which clearly indicates that LRRK2 inhibits DA release through its kinase activity. The discrepancy between the genetic and pharmacological approaches would indicate that constitutive genetic deletion of LRRK2 is not equivalent to the acute pharmacological blockade of its kinase activity, further suggesting that LRRK2 can affect DA release also via kinase‐independent mechanisms.

**Table 2:** Synopsis of the impact of genetic and pharmacological manipulation of LRRK2 on neurotransmitter release from striatal and cerebrocortical synaptosomes

	Striatal [^3^H]‐DA			Striatal GLU			Cortical GLU		
	K^+^	+ IN‐1	+ GSK	K^+^	+ IN‐1	+ GSK	K^+^	+ IN‐1	+ GSK
**WT**		**↔**	**↓**		**↓**	**↑**		↓*	**↓**
**KO**	**↑**	**↔**	**↔**	**↔**	**↔**	**↔**	**↔**	↔*	**↔**
**KD**	**↑**	**↔**	**↔**	**↔**	**↔**	**↔**	**↔**	n.t.	**↔**
**G2019S KI**	**↔**	**↑**	**↑**	**↔**	**↔**	**↔**	**↔**	n.t.	**↓**

The neurochemical effects induced by the constitutive deletion of LRRK2 (KO mice), or the expression of LRRK2 bearing the kinase‐dead D1994S mutation (KD mice) or the kinase‐enhancing G2019S mutation (KI mice) are compared with those induced by acute blockade of LRRK2 kinase activity with IN‐1 and GSK2578215A (GSK) in these preparations

↓ inhibition, ↑ increase, ↔ no change, n.t. not tested.

* 34

However, both LRRK2 inhibitors elevated the stimulus‐evoked DA release in G2019S KI synaptosomes. This suggests that the enhancement of LRRK2 kinase activity observed in this genotype 24, 25 brings the exocytotic pathways subserving DA release under a LRRK2 kinase inhibitory control, making them more sensitive toward LRRK2 kinase inhibitors. The finding that IN‐1 and GSK2578215A increased DA release at the same low concentration (0.1 μmol L^−1^) is in line with their similar high potencies in inhibiting G2019S LRRK2 (6 nmol L^−1^ vs 8.9 nmol L^−1^, respectively). Curiously, the effect in G2019S KI synaptosomes was evident only at 0.1 μmol L^−1^ but not higher concentrations, giving the concentration‐response curve a bell‐shaped profile. This profile, evident also when striatal GLU release was studied, cannot be easily explained, although it might account for the ineffectiveness of IN‐1 and GSK2578215A, tested at the single concentration of 1 μmol L^−1^, on DA release in striatal slices from hG2019S overexpressors.^23^ LRRK2 can affect neurotransmitter release via multiple routes (see Introduction). However, activation of these pathways can lead to opposite effects; in fact, LRRK2 inhibits exocytosis through snapin phosphorylation 18 and enhances it by phosphorylating the β3 subunit of the presynaptic Ca_v_2.1 calcium channel, thus increasing presynaptic Ca^++^ influx.^20^ It is therefore conceivable that the net effect on exocytosis depends on the relevance and the balance of these pathways in a specific nerve terminal as well as on the relative inhibition of these pathways achieved at different LRRK2 inhibitor concentrations.

In line with previous study with IN‐1,^34^ GSK2578215A reduced the stimulus‐evoked GLU release from cerebrocortical synaptosomes, an effect possibly associated with a reduction in vesicle trafficking.^34^ Since both IN‐1 34 and GSK2578215A were ineffective in LRRK2 KO and KD mice, these data support a facilitatory role of LRRK2 on cortical GLU exocytosis.^34^, ^48^ However, no difference in GLU release was observed in KO and KD synaptosomes when stringently compared to WT controls. This is in agreement with a study in cortical neurons obtained from KO mice 48 but in contrast with the facilitation of GLU transmission observed in cortical neurons where LRRK2 silencing was obtained via short hairpin,^16^ perhaps suggesting that constitutive or acute inhibition or LRRK2 expression may have different impact on exocytosis. Interestingly, in the presence of G2019S LRRK2, a loss of GSK2578215A potency and efficacy was observed. This would suggest that the G2019S mutation attenuates the LRRK2 facilitatory control over GLU release at cortical nerve terminals.

Different from cortex, IN‐1 and GSK2578215A oppositely modulated the stimulus‐evoked GLU release in striatal WT synaptosomes, being ineffective in KO and KD synaptosomes. This confirms that their effects rely on ongoing LRRK2 kinase activity. We do not have an easy explanation for such an opposite behavior. However, considering that IN‐1 and GSK2578215A have a different ability to inhibit LRRK2 in A2016T mutants (see Introduction) we could speculate that the different conformational changes of LRRK2 or the different levels of its kinase activity achieved at increasing LRRK2 inhibitor concentrations might result in inhibitor‐specific patterns of interactor recruitment and, therefore, presynaptic effects. Interestingly, both inhibitors failed to affect GLU release in G2019S KI synaptosomes, suggesting that GLU release from cortico‐striatal terminals in G2019S KI mice is not under, or escapes from, LRRK2 control. Consistently, striatal glutamatergic transmission is unaltered in G2019S KI mice 49, 50 (but also see 51), and GSK2578215A does not alter glutamate release 50 in G2019S KI mice.

Endogenous LRRK2 levels were reduced in the striatum and cortex of KD mice,^21^ and such reduction was observed both in synaptosomes and tissue lysates, indicating pre and postsynaptic LRRK2 pools were equally affected. Another study in a mouse carrying a different kinase‐dead mutations of LRRK2 (eg D2017A), however, reported no changes of LRRK2 levels 49 suggesting that such mutation does not cause protein destabilization. Quite surprisingly, a reduction in LRRK2 levels was also found in G2019S KI mice, although the effect was mild and area‐dependent, being significant in striatum but not in cortex. This finding contrasts with the lack of changes 25, 49 or the trend for a reduction 52 of striatal LRRK2 protein levels reported in G2019S KI mice, and should be considered when discussing biological discrepancies among different G2019S KI strains. pSer1292 levels were elevated in mice carrying the kinase‐enhancing G2019S mutation 24, 52 and undetectable in mice carrying the kinase‐silencing D1994S mutation (KD mice), confirming the validity of pSer1292 as a readout of LRRK2 kinase activity. However, given the low pSer1292 signal in WT mice,^24^, ^52^ target engagement was indirectly assessed via pSer935 levels. The reduction in pSer935 levels in G2019S KI mice might be related to the reduction in LRRK2 protein levels since LRRK2 is not hyperphosphorylated at Ser935 in this genotype.^52^, ^53^ The residual pSer935 levels seen in KD mice, where LRRK2 autophosphorylation is absent, would confirm that Ser935 phosphorylation is mediated by upstream kinases, and pSer935 levels do not rely solely on LRRK2 kinase activity. Target engagement analysis revealed differences between LRRK2 inhibitors. In fact, while both IN‐1 and GSK2578215A inhibited pSer935 levels in striatum, only IN‐1 did it in the cortex. Although the effects on neurotransmitter release and pSer935 levels cannot be directly compared since neurotransmitter release is nerve terminal specific whereas pSer935 levels are measured in the whole nerve terminal population, it is noteworthy that pSer935 changes appeared early after LRRK2 inhibitor application (3 minutes) but vanished by the time their effects on neurotransmitter release were recorded (12 minutes). This confirms that Ser935 dephosphorylation occurs very early and upstream of the cascade of events leading to neurochemical or behavioral changes.^43^ Moreover, although an overlap between the concentrations effective on neurotransmitter release and pSer935 levels was found in some case, IN‐1 appeared 100‐fold more potent in reducing pSer935 levels than neurotransmitter release in striatal synaptosomes. The potency of IN‐1 in inhibiting striatal pSer935 was remarkably high, approximating its IC_50_ for LRRK2 activity measured with an enzymatic assay (13 nmol L^−1^),^39^ much higher than that reported to inhibit pSer935 in cell lines (1‐3 μmol L^−1^).^38^, ^39^ Dephosphorylation at Ser935 has been validated as a readout of LRRK2 kinase activity in cells or ex‐vivo 36, 39, 43, 54 but has never been measured in a specific neuronal compartment (ie the nerve terminal), and particularly in a preparation of nerve terminals subjected to various preparative steps. Moreover, we should consider that Ser935 is a heterophosphosite, being phosphorylated by other kinases activated by LRRK2, or dephosphorylated by protein phosphatase 1 (PP1), which, in turn, is inhibited by LRRK2.^55^ Therefore, the potencies of Ser935 dephosphorylation in the different areas might depend on the levels and availability of endogenous LRRK2 and any other components of the pathways regulating Ser935 LRRK2 phosphorylation (which are a property of a specific nerve terminal) but also on the protocol of synaptosomes preparation, during which such components might be lost. Nonetheless, the ineffectiveness of GSK2578512A on cortical pSer935 levels corroborates a study where systemic GSK2578215A was unable to dephosphorylate brain LRRK2 at Ser935, as measured in a whole brain preparation (where the cerebrocortical tissue is predominant).^38^

In conclusion, IN‐1 and GSK2578215A exert differential effects on exocytosis and pSer935 levels in synaptosomes, which are specifically mediated by LRRK2 kinase activity but vary depending on the concentration, the nerve terminal and brain area considered. This suggests that LRRK2 inhibitors might possess unique patterns of neurochemical effects, which might rely on different modes of interaction with presynaptic LRRK2 (and associated interactors) or with different, nerve‐specific presynaptic pools of LRRK2. Expression of the G2019S mutation increases the sensitivity of DA nerve terminal to the favorable, DA‐release promoting action of LRRK2 inhibitors, at the same time attenuating their impact over GLU nerve terminals. Although these data need to be translated in vivo, they might predict a beneficial effect of LRRK2 inhibitors on DA release and therefore on motor symptoms, in G2019S LRRK2 PD patients. Likewise, the GLU‐inhibiting action of GSK2578215A in cerebrocortical synaptosomes of G2019S KI mice might also translate into a therapeutic action in view of the pathogenic role of abnormal cortical transmission in nonmotor symptoms, for example, pain and depression,^56^, ^57^ and levodopa pharmacotherapy, for example, dyskinesia,^58^ associated with PD.

## CONFLICT OF INTEREST

DRS is employed by Novartis Pharma. All other Authors declare no competing interests.

## AUTHOR CONTRIBUTION

DM and CAP prepared synaptosomes, designed and performed Western blot experiments and analyzed data. PB, MF, CAP and FL prepared synaptosomes, designed and performed release experiments and analyzed data. DRS drafted the manuscript, MM conceived the study, supervised the experiments and drafted the manuscript. All Authors revised the manuscript.
