# Supplementary material for: Leucine‐rich repeat kinase 2 (LRRK2) inhibitors differentially modulate glutamate release and Serine935 LRRK2 phosphorylation in striatal and cerebrocortical synaptosomes
Source: Pharmacol Res Perspect. 2019 May 27;7(3):e00484. doi: 10.1002/prp2.484 (PMC6536420; doi:10.1002/prp2.484)
Supplement: Supplementary file 1 [file PRP2-7-e00484-s001.pdf]

LRRK2 inhibitors differentially modulate glutamate release and Serine935 LRRK2 phosphorylation in striatal and cerebrocortical synaptosomes

#Daniela Mercatelli[1,2], #Paolo Bolognesi[1,2], #Martina Frassineti[1,2], Clarissa A. Pisanò[1,2], Francesco Longo[1,2,**], Derya R. Shimshek[3], Michele Morari[1,2,*]

[1]*Department of Medical Sciences, Section of Pharmacology, University of Ferrara, via Fossato di Mortara 17-19, 44122 Ferrara, Italy*
[2]*Neuroscience Center and National Institute of Neuroscience, University of Ferrara, via Fossato di Mortara 17-19, 44122 Ferrara, Italy*
[3]*Department of Neuroscience, Novartis Institutes for BioMedical Research, Novartis Pharma AG, 4002 Basel, Switzerland.*

#These Authors contributed equally to this work
* to whom correspondence should be addressed at Department of Medical Sciences, Section of Pharmacology, University of Ferrara, via Fossato di Mortara 17-19, 44122 Ferrara (Italy)

**Current address: Center for Neural Science, New York University, 4 Washington Place, New York, NY 10003, USA

Supplementary Material

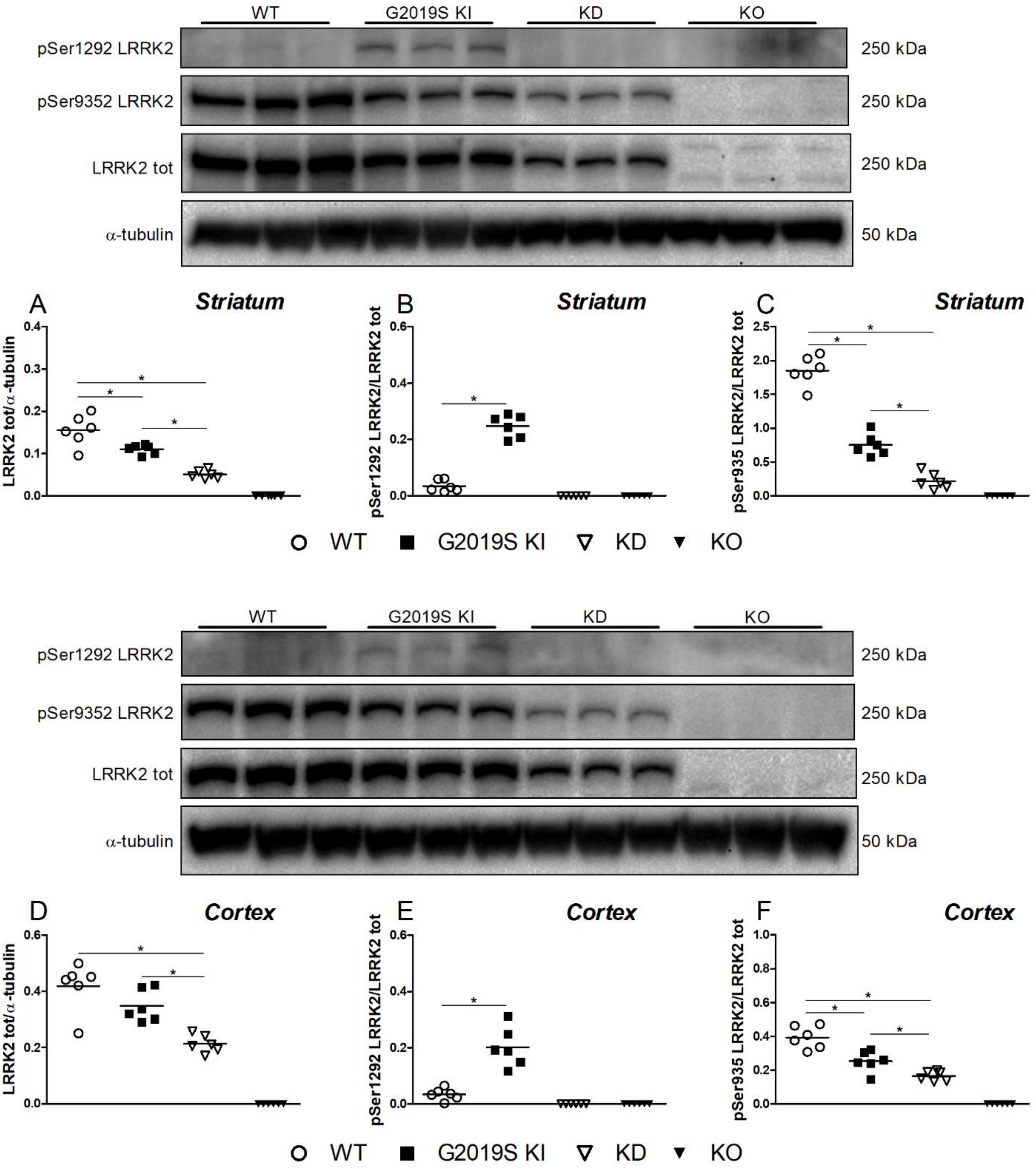

Supplementary Figure 1. Representative immunoblots and total LRRK2 (A,D), pSer1292 (B,E), and pSer935 (C,F) levels in striatal and cortical tissue lysates from WT, KD, KO and G2019S KI mice. Data are means ± SEM of 6 determinations per genotype. Each determination represents the mean of two technical replicates. *$p<0.05$ (Student's t-test or one-way ANOVA followed by the Bonferroni test)

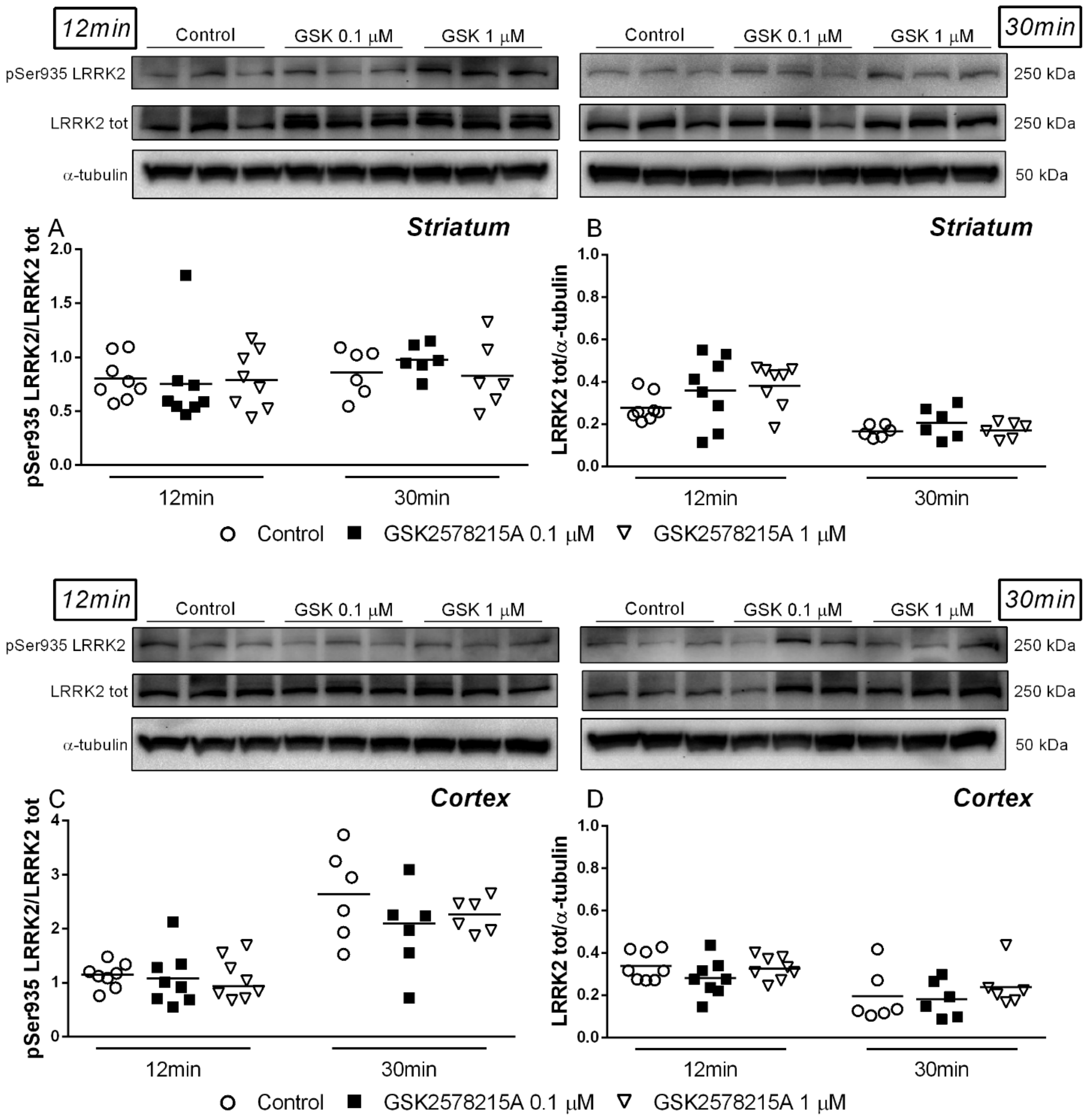

Supplementary Figure 2. Representative immunoblots, pSer935 LRRK2 (A,C) and endogenous LRRK2 (B,D) levels in striatal and cortical synaptosomes from WT mice superfused with GSK2578215A (0.1 and 1 μM) for 12 min or 30 min. Data are means ± SEM of 8 determinations (12 min) or 6 determinations (30 min) per group. Each determination represents the mean of two technical replicates.
